# Changes in male sexuality after urologic cancer: a narrative review

**DOI:** 10.1590/S1677-5538.IBJU.2023.9901

**Published:** 2022-12-15

**Authors:** Rodrigo Barros, Luciano A. Favorito, Bruno Nahar, Ricardo Almeida, Ranjith Ramasamy

**Affiliations:** 1 Hospital Universitário Antônio Pedro Universidade Federal Fluminense Niterói RJ Brasil Serviço de Urologia, Hospital Universitário Antônio Pedro, Universidade Federal Fluminense - UFF, Niterói, RJ, Brasil;; 2 Unidade de Pesquisa Urogenital Universidade Estadual do Rio de Janeiro Rio de Janeiro RJ Brasil Unidade de Pesquisa Urogenital, Universidade Estadual do Rio de Janeiro – UERJ, Rio de Janeiro, RJ, Brasil;; 3 Desai Sethi Urology Institute University of Miami Miller School of Medicine Miami FL USA Desai Sethi Urology Institute, University of Miami Miller School of Medicine, Miami, FL, USA

**Keywords:** Urologic Neoplasms, Sexual Behavior, Male

## Abstract

**Objective:**

To describe the most common sexual problems and changes experienced by male urological cancer survivors, focusing on evidence-based practices for assessment and intervention.

**Materials and Methods:**

We search the PubMed, Embase, and SciELO databases between 1994 and 2022, using the following key words: “urological cancer”, “urological malignances”, “genitourinary cancer”, “male sexual health”, and “male sexual dysfunction”.

**Results:**

This narrative review provides an overview of the current literature involving the impact of diagnosis and treatment of urological cancers on male sexual function. Male “genital” or “reproductive” tumors, such as prostate, penile, and testicular tumors, clearly appear to affect sexual function. However, tumors that do not involve genital parts of the body, such as the bladder and kidney, can also affect male sexual function.

**Conclusion:**

Male sexual dysfunction is very common after urologic cancer diagnosis and treatment. Changes in body image and anatomical damage can be associated with impaired masculinity and sexual function, especially after prostate, penile or testicular cancer treatment. Moreover, anxiety, depression, and fear of recurrence have an impact on quality of life and sexual function regardless of the cancer location. Therefore, patients need be counseled about the likely changes in sexual function before treatment of any urological cancer.

## INTRODUCTION

Sexual function is an important component of quality of life and can be adversely impacted by cancer and its treatment. Moreover, the fear of death, along with psychological and social factors, often deeply affects the quality of life of cancer patients ( 1 ).

Treatment of urological cancers can have especially significant impacts on sexual function, body image, well-being, and mental health ( 2 , 3 ). Most studies of male sexual dysfunction after urologic cancer focus on prostate cancer (PCa) survival after surgical and hormonal treatments ( 4 , 5 ). However, cancers that do not involve parts of the body designated as ‘‘sexual’’ or ‘‘reproductive’’, such as kidney (KC) and bladder cancer (BC), can also affect sexuality independent of the treatment, and their relation to sexual function is poorly understood ( 6 , 7 ).

Sexual function is a critical quality-of-life predictor and, as such, should be addressed during the treatment of all urological malignancies ( 8 ). Professionals working in this field should be aware of the impact of cancer on male sexuality. Therefore, it is important to address these topics in the urological literature. In this review, we describe the most common sexual problems and changes experienced by male urological cancer survivors, focusing on evidence-based practices for assessment and intervention.

## MATERIAL AND METHODS

We analyzed published papers contained in the PubMed, Embase, and SciELO databases between 1994 and 2022, searching by the following key expressions: “urological cancer”, “urological malignances”, “genitourinary cancer”, “male sexual health”, and “male sexual dysfunction”. Special emphasis was given to relevant articles reporting the changes in sexual health of men with urological cancers, such as prostate, penis, testicular, bladder, and kidney cancers. In this search, we included only papers published in English and excluded case reports, editorials, and opinions of specialists.

## RESULTS

This narrative review provides an overview of the current literature involving the impact of diagnosis and treatment of urological cancers on male sexual function. Male “genital” or “reproductive” tumors, such as prostate, penile, and testicular tumors, clearly appear to affect sexual function. However, tumors that do not involve genital parts of the body, such as the bladder and kidney, can also affect male sexual function.

### Prostate cancer (PCa)

PCa is the second most often diagnosed cancer among men worldwide ( 9 ). Different treatment modalities for PCa can negatively affect sexual function. Surgery is the reference standard for treatment of localized PCa. Nerve-sparing radical retropubic prostatectomy was developed many years ago to preserve sexual potency and urinary continence. Catalona et al. ( 10 ) evaluated the results of 1,870 open retropubic prostatectomies (ORP) performed by a single surgeon and found recovery of erectile function in 68% of preoperatively potent men treated with bilateral (543 of 798) and 47% treated with unilateral (28 of 60) nerve sparing surgery. Today, minimally invasive techniques such as laparoscopic radical prostatectomy (LRP) and robotic-assisted laparoscopic radical prostatectomy (RALP) have replaced ORP to improve post-operative outcomes such as erectile function ( 11 ). Guillonneau et al. ( 12 ) evaluated their experience with 550 patients who underwent LRP and found that 66% preserved erection and could engage in spontaneous intercourse. Patel et al. ( 13 ) analyzed the initial outcomes of 500 RALPs and found that after one year, 78% of patients were potent with or without the use of oral medications. More recently, Barisi et al. ( 4 ) conducted a systematic literature review comparing ORP, LRP, and RALP, where one of the outcomes was erection dysfunction (ED). According to this study, there were no differences in post-operative rates of ED between ORP and LRP or RALP. Interestingly, LRP was associated with greater post-operative rates of ED when compared with RALP. However, this review should be interpreted with caution due to the lack of randomized clinical trials, selection bias, and heterogeneous definitions of ED. In addition to ED, sexual changes after radical prostatectomy include loss of penile length, reduced sexual desire, and orgasmic dysfunction, including painful orgasm and climacturia, or involuntary loss of urine at the time of orgasm ( 14 - 16 ). True rates of climacturia are unknown and probably underreported in the literature ( 17 ). Clavell-Hernandez et al. ( 18 ) conducted a review of the literature on climacturia after radical prostatectomy and found prevalence ranging from 20% to 93%.

ED after radiotherapy (RT) usually occurs due to penile neurovascular and cavernosal damage. While ED is an immediate side effect of radical prostatectomy, it usually occurs after six months post radiation therapy. Donovan et al. ( 5 ) report that only 22% of men-maintained erections firm enough for intercourse six months after RT with neoadjuvant androgen deprivation therapy (ADT). Likewise, Kikuchi et al. ( 19 ) evaluated erectile function after RT in 55 patients with PCa and observed a decrease in the erectile function and intercourse satisfaction after RT. Another study evaluated sexual functions of 50 PCa patients receiving RT. The authors used the IIEF (International Index of Erectile Function) questionnaire before and on the last day of treatment. They found a statistically significant decline in erectile function, sexual desire, sexual satisfaction, orgasmic function, and general satisfaction after RT. Considering that ED is usually chronic side effect of RT, these findings might reflect a psychological side effect of RT ( 20 ).

While radical treatment with surgery or radiation offers excellent cancer control, it comes with significant side effects as discussed previously. Alternative treatments with less impact in quality of life and sexual function have gained popularity in recent years.

Focal therapy (FT) is an a less invasive option that treats only the cancerous area of the prostate (aka index lesions) and maintains patient’s quality of life by avoiding some of the adverse effects of radical therapy, including ED. Several studies with large sample size and long follow up showed benefits of FT on functional outcomes ( 21 - 24 ). Nahar et al. ( 22 ) reported short-term outcomes of FT for primary treatment of localized PCa and observed that sexual function returned to baseline at within 9-12 months. Similarly, Rischmann et al. ( 23 ) evaluated 111 patients with unilateral localized PCa treated with high intensity focused ultrasound (HIFU). Erectile function was preserved in 78% of patients after 12 months of HIFU half-gland treatment. A recent study compared the impact of focal (N = 195) and whole gland (N = 105) therapy for PCa on erectile and urinary function. Twelve months after treatment, 81.3% of men who underwent FT (vs. 61.7% of whole gland patients) could achieve erection strong enough for sexual penetration ( 24 ).

Similarly, Active surveillance (AS) is one the preferred choice for patients with low-risk prostate cancer. However, even men under AS can suffer negative impacts on sexual function. Soloway et al. ( 25 ) followed men in AS for PCa and observed 49% of patients experiencing ED. Another study compared the sexual function of men with low-risk PCa monitored through AS with patients undergoing RT or radical prostatectomy and found that the AS group had less ED ( 26 ).

Patients with metastatic prostate cancer are usually treated with androgen deprivation therapy (ADT) with the goal of reducing serum testosterone levels. Therefore, castration levels of testosterone results in multiple side effects, including loss of libido and ED. It’s extremely important to correctly inform patients about these well-known side effects before starting treatment ( 27 , 28 ).

### Penile cancer (PEC)

PEC is rare in North America and Europe; the incidence is higher in regions of Africa, Asia, and South America due to due socioeconomic factors and the high incidence of the human papilloma virus (HPV), phimosis, and smoking in these regions ( 29 - 31 ). The treatment modalities of PEC depend on the area involved and include some organ-sparing treatments such as topical therapy, laser therapy, RT, glansectomy, wide-local excision, and partial penectomy. Total penectomy is reserved for cases with more advanced primary disease ( 32 ).

All types of treatment for PEC can impact quality of life and sexual function. Glansectomy seems to preserve sexual function by maintaining the ability to perform vaginal penetration and leaving libido and ejaculation function intact; however, the few studies available evaluating the results of the procedure had small sample sizes and several methodological flaws ( 33 - 35 ). Palminteri et al. ( 36 ) described the techniques and results of surgical reconstruction of glans penis lesions (benign, premalignant, and malignant). In their series, five cases were treated with glans resurfacing, five glansectomies with neoglans reconstruction were performed, and seven patients underwent partial penectomy and reconstruction of the neoglans. All patients maintained sexual function and activity. Patients who underwent glans resurfacing reported glandular sensory restoration while sensitivity was reduced after glansectomy and partial penectomy. Partial or total penectomy can be associated with significant psychological morbidity and sexual dysfunction. Feelings of shame due to the small penis size and the absence of the glans are some reasons for the negative impact on male sexual function. In one such study, Romero et al. ( 37 ) investigated 18 patients who underwent partial penectomy and reported a statistically significant reduction in erectile and orgasmic function after surgery. According to the authors, only 33.3% of patients maintained their preoperative sexual intercourse frequency and were satisfied with their overall sex life after the procedure. Monteiro et al. ( 38 ) evaluated the erectile function of 81 patients who underwent partial penectomy and reported that approximately 62% experienced ED after surgery. The authors found that smaller penile shaft length, clinically positive lymph node, and older age significantly increased the incidence of ED. In the study conducted by Opjordsmoen et al. ( 39 ), four of 30 men treated for PEC underwent total penectomy, and all of them reported severely reduced global sexual score. Due to the rarity of PEC, there are few studies available exploring sexual outcomes after treatment. Although most of the papers are retrospective with a small sample, it is clear that an penile malignancies and treatments negatively impact patients’ sexuality. Therefore, physicians should counsel patients with this rare malignancy about the impact and changes of male sexual function that they are likely to experience after PEC treatment. Referral to psycho-oncology might be beneficial to patients.

### Testicular cancer (TC)

TC accounts for about 1% of all male cancers and characteristically affects mostly young men (aged 20–40 years). TC has a good prognosis with excellent cure rates in the early stages when treated by one of the standard treatment options, including orchiectomy, RT, and cisplatin-based chemotherapy ( 40 , 41 ). Treatment of TC can cause changes in body image and negatively impact sexuality, fertility, mental health, and quality of life. An Australian study found that TC survivors experienced anxiety and depression in 19% and 20% of cases respectively ( 42 ). Rincones et al. ( 43 ) conducted a systematic review of anxiety, depression, fear of cancer recurrence and distress in TC survivors. The authors concluded that greater anxiety and depression seemed to be associated with impaired masculinity, sexual function, and quality of life. Changes in body image after orchiectomy can impact self-confidence and sexuality, and it is extremely important that physicians offer a testicular prosthesis implant at the time of surgery ( 44 ). A systematic review conducted by Nazareth et al. ( 45 ) of sexual dysfunction in men treated for TC indicated significantly reduced or absent orgasm and ejaculatory dysfunction that persisted for up to two years after treatment. Not surprisingly, ejaculatory dysfunction was most frequently related to retroperitoneal lymph node dissection (RPLND) surgery ( 46 ). Palotti et al. ( 47 ) evaluated the possible effect of TC and orchiectomy on sexual function. They administered the IIEF-5 to TC patients at the post-orchiectomy baseline before chemotherapy and found that 37.7% of patients had ED. According to the authors, the sexual dysfunction in these patients might be associated with psychological burden. In fact, sexual dysfunction in TC is not clearly related to disease or treatment factors and may instead arise from psychological vulnerability ( 46 ).

### Bladder cancer

Bladder cancer (BC) is the fifth most common cancer in men worldwide ( 48 ). Most patients have non-muscle-invasive bladder cancer (NMIBC), which is commonly treated with transurethral resection of bladder tumor (TURBT). There is scarce research on the effect of treatment for NMIBC on male sexual function. Existing research suggests that TURBT may adversely affect male sexuality and lead to anxiety and depression, especially in younger patients ( 49 ). Guo et al. ( 7 ) investigated the incidence of ED in patients before and after TURBT to treat NMIBC. According to the authors, the incidence of ED increased in patients under the age of 45 years after TURBT (15.8% before vs. 52.6% after), and they concluded that psychological and emotional burden are the main causes of sexual dysfunction in these cases. Yoshimura et al. ( 50 ) prospectively evaluated the impact on general health-related quality of life of patients with NMIBC who underwent TURBT. They found physical and mental problems after the first TURBT, but these problems gradually waned as TURBT was repeated, although the patients’ general quality of life remained affected. More than a half of NMIBC cases will recur and intravesical bacille Calmette–Guérin (BCG) treatment has an important role in reducing this recurrence ( 51 ). Patients who received intravesical BCG might present with pelvic pain and may experience a negative impact on sexual activity after the initial treatment. Nonetheless, patients improved their psychological distress and physical symptoms as they continued the treatment ( 52 , 53 ). ED after BCG treatment is generally transient and reversible but is still another source of psychological distress ( 54 ). Radical cystectomy (RC) remains the gold standard treatment in cases of muscle invasive bladder cancer (MIBC). It consists of removal of the bladder, prostate, and seminal vesicles ( 55 ). ED after RC is a prevalent problem due to surgical trauma to the neurovascular bundle, and one study found that only 14% of sexually active men-maintained potency after surgery ( 56 ). However, nerve-sparing RC can often provide preservation or recovery of erectile function, and 36% of RC patients recovered sexual intercourse at 3 years and 57% at 5 years. This recovery depends on the preoperative erectile function and age of the patient. Function can be improved after sexual rehabilitation with intracavernous injection therapy or oral phosphodiesterase inhibitors after surgery ( 57 , 58 ). The type of urinary diversion can also affect sexual activity. Patients with ileal conduit diversion may have a greater impact on sexual function compared to those who underwent orthotopic diversion likely due to depression or anxiety associated with changes in body image ( 59 ). Trimodality therapy (TMT) can be used as an alternative to immediate RC in the management of MIBC. TMT consists of maximal TURBT followed by radical RT with concurrent chemotherapy ( 60 ). Radical RT for BC can result in sexual dysfunctions such as impotence and lack of desire ( 61 ). Zietman et al. ( 62 ) performed a small retrospective study of TMT and found male sexual function to be less impaired by this modality than after RC. A total of 39% of men reported no erections in the last 4 weeks, 54% were capable of orgasm and 50% of ejaculation, while only 8% were dissatisfied with their sex lives.

### Kidney cancer (KC)

KC incidence is increasing, and over 50% of KC tumors are diagnosed incidentally in asymptomatic individuals during investigation for other conditions using imaging techniques ( 63 , 64 ). The literature is scarce about the impact on male sexual function after treatment for KC. Anastasiadis et al. ( 65 ) published the first study addressing sexual function in patients with KC after treatment (operation, radiation, or chemotherapy). They observed that most patients remained sexually active in non-distressed relationships, but 51% of men reported depressive symptoms, and sexual functioning may be worse than in comparable chronically ill populations. Christiansen et al. ( 6 ) evaluated patients who underwent nephrectomy or nephroureterectomy and found that 54.7% of sexually active males reported having some degree of ED after surgery. Moreover, 61% of patients reported being worried about their sex lives. Interestingly, only 5% of patients were informed about these potential negative effects prior to surgery. Few studies have investigated sexual disorders in men with advanced KC treated with molecular targeted therapy (MTT); antiangiogenic therapies (sunitinib, sorafenib, and bevacizumab) and mTOR inhibitors (temsirolimus and everolimus) caused a decline of erectile function scores and sexual activity after treatment ( 66 , 67 ). These studies concluded that treatment of KC can negatively affect male sexual function. The diagnosis of cancer, life stress, and losses can explain the sexual dysfunction after treatment, which is information that should be provided to patients ( 1 , 68 ). Table-1 summarizes the risk of ED after type of urologic cancer treatment.

**Table 1:** Risk of ED after type of urologic cancer treatment.

Study	Year	Treatment	Risk of ED
Catalona et al. ( 10 )	1999	ORP	32%
Guillonneau et al. ( 12 )	2002	LRP	34%
Patel et al. ( 13 )	2007	RALP	22%
Donovan et al. ( 5 )	2016	RDT + NEOADJUVANT ANDROGEN THERAPY	78%
Borges et al. ( 24 )	2021	FOCAL HIFU	18%
Soloway et al. ( 25 )	2010	AS	49%
Monteiro et al. ( 38 )	2021	PARTIAL PENECTOMY	62%
Guo et al. ( 7 )	2022	TURBT	56%
Palotti et al. ( 47 )	2019	ORCHIETOMY	37%
Zippe et al. ( 26 )	2004	RC	86%
Miyao et al. ( 57 )	2001	NERVE-SPARING RC	43%
Zietman et al. ( 62 )	2003	TMT	39%
Christiansen et al. ( 6 )	2020	NEPHRECTOMY OR NEPHRO-URETERECTOMY	54%

## CONCLUSIONS

Male sexual dysfunction is very common after urologic cancer diagnosis and treatment. Changes in body image and anatomical damage can be associated with impaired masculinity and sexuality, especially after PCa, PEC, or TC treatment. Moreover, anxiety, depression, and fear of recurrence have an impact on quality of life and sexual function even in “nonreproductive” cancers, such as BC and KC.

Therefore, patients need be counseled about the likely changes in sexual function before treatment. Urologists and oncologists should systematically inform, educate, and comfort these patients during the treatment. Multidisciplinary medical teams, including sexual medicine physicians and psycho-oncologist, play a fundamental role in this scenario and need to be proactive by offering psychological support to mitigate the impact on male sexuality. However, more studies are needed to clarify the impact urological malignances and their treatments may have on the sexual function of men, and clinicians need better training about the best way topproach these issues.
